# Plasticity of Non-Apoptotic Residual Tumor Cells After Neoadjuvant Immunochemotherapy: Epigenetic and Microenvironmental Determinants

**DOI:** 10.3390/biom16071065

**Published:** 2026-07-21

**Authors:** Wenjun Meng, Ruiyue Li, Peiliang Xie, Bangyi Xiang, Qing Li

**Affiliations:** 1 Department of Pain Management, West China Hospital, Sichuan University, Chengdu 610041, China; mwj1995@scu.edu.cn (W.M.);; 2 Outpatient Department, West China Hospital, Sichuan University, Chengdu 610041, China; 3 West China School of Medicine, Sichuan University, Chengdu 610041, China; 4 Department of Biotherapy, Cancer Center, West China Hospital, Sichuan University, Chengdu 610041, China; 5 Department of Biotherapy, Cancer Center, National Clinical Research Center for Geriatrics, West China Hospital, Sichuan University, Chengdu 610041, China

**Keywords:** neoadjuvant therapy, plasticity of non-apoptotic residual tumor cells, residual disease, drug-tolerant persister cells, epigenetic regulation, tumor microenvironment, microenvironmental niches

## Abstract

Neoadjuvant immunochemotherapy (NICT), mainly anti-PD-1/PD-L1 therapy combined with cytotoxic chemotherapy, significantly improved perioperative outcomes for resectable solid tumors such as lung cancer and breast cancer. But a large number of patients still had residual lesions and eventually relapsed. Residual tumor cells are not simply unremoved cellular debris, but represent a therapy-selected and therapy-amplified subset of a pre-existing heterogeneous and plastic tumor ecosystem. To avoid implying that therapy generates a new form of tumor plasticity de novo, we use the term “plasticity of non-apoptotic residual tumor cells” to describe the plastic behavior of viable malignant cells that survive treatment-induced cytotoxicity rather than entering apoptosis. In this review, we define the plasticity of non-apoptotic residual tumor cells as the capacity of residual malignant cells to preserve, switch, or re-enter phenotypic states such as dormancy, hybrid EMT, stem-like regeneration, and immune evasion under the combined influence of intrinsic tumor heterogeneity, systemic therapy pressure, and microenvironmental protection. Before the NICT-specific discussion, we outline general theoretical frameworks including therapeutic stress response, apoptosis-induced regeneration, genetic and non-genetic heterogeneity, as well as spatial heterogeneity of involved lymph nodes, so as to provide a more robust interpretation of residual lesion biology under NICT. Also, this review proposes that residual disease may be reconceptualized as a treatment-shaped plastic niche, whose biological behavior is jointly shaped by clonal selection, reversible phenotypic transformation, and microenvironmental ecological protection. We summarize several key states of residual tumor cells: persistent-like/resting state, hybrid EMT/invasive plasticity state, stem-like/regenerative state, and immune escape state, and elucidate the underlying epigenetic basis, including DNA methylation, histone modification, chromatin remodeling, and non-coding RNA network reprogramming. Meanwhile, niche factors such as immune stress, CAF/TAM enrichment, fibrotic matrix, hypoxia, and metabolic stress can further stabilize these states and promote the survival of relapse seeds. Based on this, we propose that future postoperative assessments should be upgraded from residual volume to a stratified residual state, and dynamically identified by combining single-cell omics, spatial pathology, and ctDNA/MRD monitoring. Furthermore, treatment strategies should shift from simply shrinking tumors to plasticity-locking therapy, that is, identifying, classifying, and blocking the plasticity escape pathways of residual lesions before they evolve into recurrence.

## 1. Introduction

### 1.1. Clinical Significance of Neoadjuvant Immunochemotherapy and Residual Disease

Neoadjuvant therapy, particularly neoadjuvant immunochemotherapy (NICT), is reshaping the perioperative treatment landscape for resectable solid tumors, especially lung cancer and breast cancer [1]. However, even with significant tumor shrinkage, a considerable proportion of patients retain residual lesions postoperatively, eventually leading to tumor recurrence or metastasis [2]. This result suggests that a residual tumor is not simply unremoved cellular debris, but rather an adaptive survival population formed after treatment selection [3,4,5]. Current pathological assessment systems primarily focus on the volume and proportion of residual tumor cells, but fail to adequately capture the functional state of residual tumor cells and their dynamic interactions with the microenvironment [6].

The aims of neoadjuvant therapy are multifaceted: to downstage tumors and improve resectability, to eradicate micrometastatic disease that may already be present at diagnosis, to assess treatment sensitivity in vivo and thereby inform postoperative management, and in the immunotherapy era, to leverage the intact tumor as an in situ antigen source for priming systemic anti-tumor immunity [7,8]. NICT, which combines cytotoxic chemotherapy with immune checkpoint inhibition, has demonstrated significant improvements in pathological complete response (pCR) rates and event-free survival (EFS) across multiple tumor types without increasing perioperative risks. In resectable non-small-cell lung cancer (NSCLC), CheckMate 816 showed that neoadjuvant nivolumab plus chemotherapy increased pCR from 2.2% to 24.0% and improved EFS (HR 0.63) [9], while the NADIM II trial reported pCR rates of 36.3% versus 6.8% with chemotherapy alone [10]. The perioperative KEYNOTE-671 regimen similarly improved EFS (HR 0.58) in stage II–IIIB NSCLC [11]. Two additional perioperative phase III trials—AEGEAN (perioperative durvalumab plus chemotherapy) and CheckMate 77T (perioperative nivolumab plus chemotherapy)—further confirmed EFS benefits in resectable NSCLC [12,13]. In early triple-negative breast cancer (TNBC), KEYNOTE-522 demonstrated that adding pembrolizumab to neoadjuvant chemotherapy increased pCR from 51.2% to 64.8% and significantly improved EFS [14]. Importantly, systematic reviews and meta-analyses have confirmed that the addition of immunotherapy to neoadjuvant chemotherapy does not significantly increase surgical complications, delay surgery, or raise perioperative mortality [15].

Whether these improvements in pCR and EFS translate into overall survival (OS) benefit has been a central question. Recent evidence has now provided affirmative answers in key settings: the 5-year OS analysis of CheckMate 816 demonstrated a significant OS benefit with neoadjuvant nivolumab plus chemotherapy (HR for death 0.72; *p* = 0.048; 5-year OS 65.4% vs. 55.0%) [9], and the final OS analysis of KEYNOTE-522 confirmed significant OS improvement in early TNBC (60-month OS 86.6% vs. 81.7%; *p* = 0.002) [16]. However, mature OS data are still awaited for other tumor types where NICT is increasingly adopted, such as esophageal cancer (EC), gastric cancer (GC), and head and neck cancer (HNC). These emerging survival data further underscore the clinical importance of understanding residual disease biology; the patients who fail to achieve pCR and retain residual lesions represent the population in whom survival gains are lost, making the study of residual tumor plasticity a direct translational imperative.

The choice to focus specifically on NICT in this review is motivated by both clinical urgency and mechanistic rationale. Since 2022, NICT has been established as standard of care for resectable NSCLC, early TNBC, and increasingly for EC, GC, and HNC [10,11,14,17]. This rapid, practice-transforming adoption means that understanding the biology of residual disease after NICT is no longer a niche academic question, but a pressing translational imperative affecting tens of thousands of patients annually.

Moreover, NICT generates a dual selection landscape that distinguishes it from other neoadjuvant cytotoxic modalities [18,19]. We do not argue that apoptosis itself differs in NICT; apoptosis is apoptosis, and the intrinsic mitochondrial, extrinsic death receptor, and cytotoxic lymphocyte-mediated pathways described in Section 1.4 are shared across chemotherapy, radiotherapy, and targeted therapy [20,21,22,23,24]. Rather, what sets NICT apart is the selective pressure that determines which cells survive; chemotherapy alone selects for drug-tolerant persisters (DTPs) through pharmacological pressure (drug efflux, DNA repair, apoptosis evasion), whereas concurrent immune checkpoint inhibition imposes an additional immune-selective dimension: epigenetically mediated downregulation of MHC-I/B2M/TAP1-TAP2, IFN-γ pathway rewiring, and upregulation of alternative immune checkpoints (PD-L1, CD47, GAL9) that is absent in chemotherapy-only, radiotherapy, or targeted therapy settings [18,24]. Direct evidence for this distinction comes from the NEOSUMIT-01 randomized trial, which identified NICT-unique resistance mechanisms; an APOA1^+^ tumor cell-TREM2^+^ macrophage immunosuppressive axis and HLA-B*15:02-associated defective antigen presentation, that were absent in chemotherapy-alone resistance [25]. This immune-selective layer, superimposed on chemotherapy-driven persister selection, constitutes the specific feature that differentiates NICT from other neoadjuvant cytotoxic modalities and makes it a particularly informative paradigm for studying therapy-induced plasticity.

Despite above improvements, residual disease remains the most common outcome after NICT. Across NSCLC, pCR is achieved in only 24–36%, leaving 64–76% with residual lesions; in EC and GC, residual disease rates are 70–85%; even in TNBC, approximately 35% of KEYNOTE-522 patients retained residual tumor [9,10,14]. This is not merely a volumetric problem, and a meta-analysis of 43 trials (4100 patients) found that non-pCR after neoadjuvant immunotherapy is associated with approximately twice the risk of an EFS event (HR for pCR vs. non-pCR 0.48) and a ~45% higher risk of death (OS HR 0.55) across solid tumor types [26]. These data establish residual disease as one of the most prevalent and prognostically significant unmet needs in oncology, and provide the quantitative impetus for the mechanistic analysis that follows. Residual disease remains the major obstacle to durable cure after NICT.

### 1.2. Tumor Plasticity and the Biological Nature of Residual Tumor Cells

Emerging evidence suggests that the biological nature of residual tumor lesions is closely related to tumor plasticity, meaning that tumor cells can dynamically switch between stem-like, epithelial–mesenchymal transition (EMT)/hybrid EMT, and quiescence/reactivation to evade immune attack and treatment stress [27]. The plasticity of cancer stem cell-like cells, hypoxia-driven state transitions, and their coupling with treatment tolerance have been recognized as important theoretical frameworks for explaining recurrence [28]. Meanwhile, longitudinal transcriptomic studies further demonstrate that drug resistance-related programs often exist before treatment, and the treatment process is more like a screening and amplification of pre-existing tolerance states [29].

In this context, it is important to clarify what we mean by the plasticity of non-apoptotic residual tumor cells. Tumor plasticity is not a phenomenon that emerges only after NICT; rather, it is an intrinsic property of malignant tissues and is closely linked to intratumoral heterogeneity, clonal diversity, and non-genetic state transitions [30]. Systemic therapy does not necessarily create plasticity de novo, but can select pre-existing tolerant clones, accelerate reversible phenotypic switching, and stabilize adaptive states through treatment-induced microenvironmental remodeling [31].

Therefore, the term “plasticity of non-apoptotic residual tumor cells” is used here as an operational descriptor, not as a new biological category of plasticity. Rather, the framework’s conceptual contribution is to integrate genetic clonal selection, non-genetic adaptive plasticity, and microenvironmental ecological protection into a unified three-axis model; whereas established residual disease frameworks have typically addressed these axes in isolation [31,32], and to extend classical chemotherapy-era residual disease biology to include the immune-evasive state uniquely selected by NICT. It specifically refers to the plastic states of viable malignant cells that remain detectable after neoadjuvant treatment and have escaped treatment-induced apoptotic elimination [18,32,33]. These non-apoptotic residual cells may persist in persister-like/dormant, hybrid EMT/invasive, stem-like/regenerative, or immune-evasive states, thereby serving as potential relapse-competent reservoirs [34,35].

### 1.3. Microenvironmental and Epigenetic Regulation of Residual Cell States

At the microenvironment level, NICT can significantly reshape the tumor immune ecology. In EC, treatment response is closely related to the density of CD3^+^, PD-L1^+^ cells in the tumor area; furthermore, a more significant increase in CD8^+^ and CD20^+^ cells was found in patients who were responsive to treatment, while non-responders were more likely to exhibit characteristics of PD-L1 upregulation and immune exhaustion [3,4,36]. These changes suggest that residual lesions are not immune silence but are in a state of continuous immune selection and reprogramming.

Meanwhile, epigenetic reprogramming provides a reversible molecular basis for residual tumor plasticity. DNA methylation, histone modification, chromatin remodeling, and non-coding RNA networks can collectively regulate antigen presentation, stem programming, EMT, metabolic adaptation, and immune escape, enabling tumor cells to acquire short-term tolerance and long-term evolutionary advantages without altering their gene sequences [27,28,37,38,39]. Therefore, understanding the plasticity of residual tumors after NICT requires discussing microenvironmental selection pressures and epigenetic memory within the same framework.

### 1.4. General Biological Principles Underlying Therapy-Induced Residual Disease

Before specifically discussing NICT, a concise and general theoretical framework needs to be established to harmonize the terminology and mechanistic concepts. The initial response of tumors to systemic therapy is typically driven by broad therapy-induced stress responses, including DNA damage and replication stress, metabolic and redox reprogramming, as well as activation of the integrated stress response (ISR) and inflammatory signaling pathways [40,41]. These stress signals can lead to either irreversible cell death or adaptive reprogramming of surviving cells through stress-adaptive programs. Second, apoptosis should not be regarded merely as an endpoint of cellular elimination. Increasing evidence indicates that apoptotic cells can actively transmit pro-regenerative signals to neighboring viable tumor cells through “Phoenix Rising”-like paracrine mechanisms, involving pathways such as caspase-3 activation followed by iPLA2-COX-2-PGE2 signaling [20,42]. These processes may promote repopulation and regrowth of residual tumor cells after therapy. Third, regarding mechanisms of tumor cell elimination, immunochemotherapy-induced cytotoxicity is mediated through multiple parallel pathways, including perforin/granzyme release by cytotoxic lymphocytes, the extrinsic apoptotic pathway mediated by death receptors (Fas/TRAIL → caspase-8), and the intrinsic mitochondrial pathway (BAX/BAK → cytochrome-c → apoptosome → caspase-9 → caspase-3) [21,22]. The interplay between these death pathways and the TME may generate distinct paracrine effects and immunological consequences. Fourth, tumor genetic heterogeneity determines differential sensitivity of distinct clones to systemic therapies through processes of clonal selection. Importantly, metastatic dissemination is now recognized as an early event in tumor evolution, such that metastatic deposits, including involved lymph nodes and skip lesions may already be genetically heterogeneous before systemic therapy is initiated [43,44]. Systemic therapy further drives both genetic and epigenetic evolution of these pre-existing heterogeneous populations through treatment-induced selective pressure and epigenetic remodeling [31,45,46]. Consequently, achieving a durable cure in the neoadjuvant setting ultimately depends on managing this intrinsic tumor heterogeneity, not merely eradicating macroscopic residual lesions. In parallel, non-genetic mechanisms, including reversible DTP states, allow tumor cells to survive transient therapeutic pressure. These two mechanisms can coexist and collectively shape the biological characteristics of residual disease [40,41]. Finally, involved lymph nodes and so-called “skip” lesions may exhibit substantial spatial heterogeneity in both genomic composition and immune microenvironment. Therefore, clinical resection strategies, pathological evaluation, and postoperative treatment decisions should account for this spatial complexity [47,48].

Apoptosis should therefore be considered not merely as the opposite state of the viable residual cells discussed, but as both an upstream therapeutic event and a niche-shaping biological process [20,23,49]. In the chemotherapy component of NICT, DNA damage, replication stress, mitotic stress, and oxidative stress can converge on the intrinsic mitochondrial apoptotic pathway, involving BAX/BAK-mediated mitochondrial outer membrane permeabilization, cytochrome-c release, apoptosome formation, caspase-9 activation, and downstream caspase-3/7 execution [20,23]. In the immune component, PD-1/PD-L1 blockade does not directly induce tumor-cell apoptosis in the same manner as cytotoxic drugs; rather, it restores cytotoxic CD8^+^ T-cell and NK-cell activity, which can eliminate tumor cells through perforin/granzyme B-mediated apoptosis and through death receptor pathways such as Fas/FasL and TRAIL-DR4/DR5, followed by FADD recruitment, caspase-8 activation, and convergence on caspase-3/7 or mitochondrial amplification [24,50]. Thus, chemoimmunotherapy may engage intrinsic mitochondrial, extrinsic death receptor, and cytotoxic lymphocyte granule-associated apoptotic programs in treatment-sensitive tumor fractions [20,23,24]. Importantly, apoptotic tumor cells may also paradoxically support tumor repopulation by releasing paracrine regenerative signals [20,49]. Executioner caspases, especially caspase-3/7, can activate iPLA2 and downstream COX-2/PGE2 production, thereby promoting Wnt/β-catenin signaling, tissue repair-like responses, and compensatory proliferation in neighboring viable cells [20,49]. Therefore, in this review, non-apoptotic residual tumor cells are defined as the viable cellular compartment that retains plastic potential after therapy, whereas apoptotic tumor cells are considered an upstream source of cytotoxic selection, paracrine regrowth signals, and residual niche remodeling.

### 1.5. Scope and Objectives of the Review

Within this general framework, this review subsequently focuses on how the unique dual selective pressures imposed by NICT, combining chemotherapy and immune-mediated killing amplify and consolidate these fundamental mechanisms, ultimately shaping the biology of residual tumor disease.

## 2. Conceptual Model: Selection, Adaptation and Ecological Protection

The conceptual framework developed in this section is anchored on NICT, which serves as a particularly instructive model because it imposes simultaneous pharmacological and immune selection pressures, thereby enriching the full spectrum of plastic states, including immune-evasive phenotypes that are not selected for by chemotherapy alone. However, the core principles of clonal selection, adaptive plasticity, and ecological protection are broadly applicable to other neoadjuvant modalities, and we note where evidence from non-NICT contexts informs the model.

Tumor plasticity is a fundamental property of malignant tissues and a major source of intratumoral heterogeneity [51,52]. It allows cancer cells to shift among proliferative, dormant, invasive, stem-like, and immune-evasive states without requiring immediate acquisition of new driver mutations. In the setting of systemic therapy, this pre-existing plastic potential becomes clinically visible because treatment eliminates sensitive populations while selecting, accelerating, or stabilizing tolerant cellular states [18].

**Figure 1:**
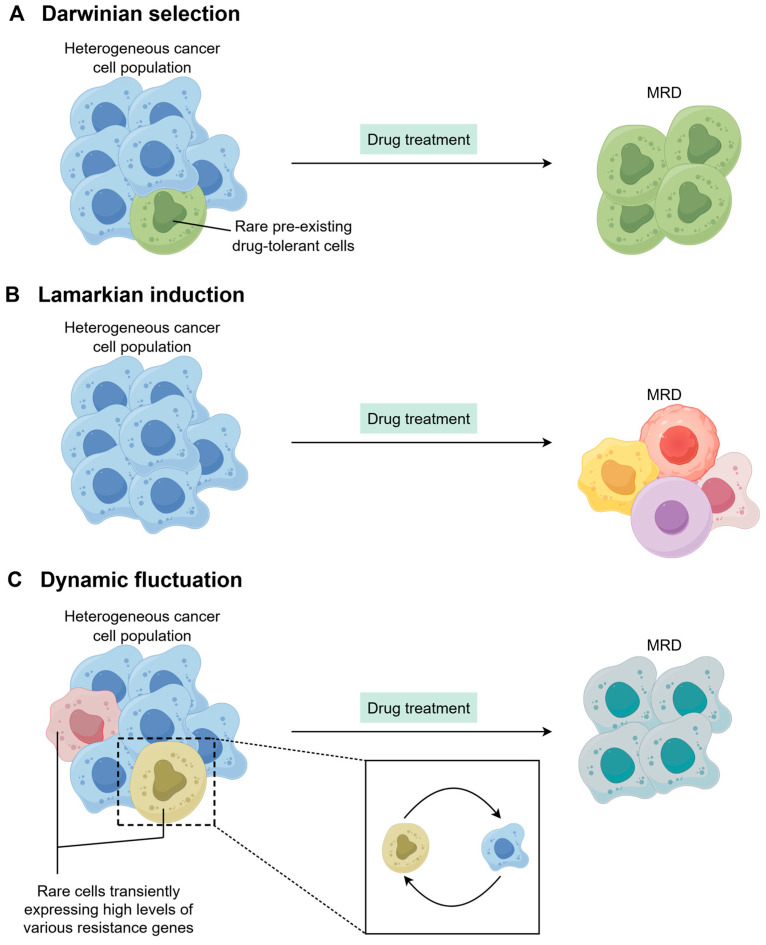
Multiple theoretical frameworks have been put forward to explain how non-apoptotic, drug-tolerant residual tumor cells persist following anticancer interventions. (**A**) The Darwinian selection model holds that a tiny subset of drug-tolerant cells already resides within tumors prior to antitumor treatment, and only this subpopulation is capable of surviving drug exposure. (**B**) According to the Lamarkian induction hypothesis, drug exposure triggers epigenetic alterations in heterogeneous cancer cell populations, which then generate drug-tolerant cell populations; multiple distinct drug-tolerant phenotypes may form simultaneously and persist together within residual tumor lesions. (**C**) The third proposed mechanism states that cancer cells display variable expression levels of resistance-related biomarkers. When treatment commences, rare tumor cells with abundant expression of diverse resistance genes manage to survive, followed by transcriptional reprogramming that locks in stable expression profiles of resistance gene signatures. Notably, these distinct models do not contradict one another and can co-occur. As one illustrative example, researchers hypothesize that pre-existing rare cancer cell subpopulations can withstand chemotherapy, then undergo differentiation to acquire a drug-tolerant phenotype. MRD, minimal residual disease.

Accordingly, the plasticity discussed in this review should be understood as the plasticity of non-apoptotic residual tumor cells: viable malignant cells that have survived NICT rather than undergoing apoptosis. These cells are not assumed to have acquired a new plastic property after therapy; instead, therapy exposes, enriches, and stabilizes pre-existing or inducible plastic states within the residual disease compartment [53,54,55]. Furthermore, treatment resistance is often not explained by a single mechanism, but rather by the combined effects of genetic selection, non-genetic adaptation, and ecological protection [45]. The distinct scenarios through which therapy selects and stabilizes residual disease are conceptualized in Figure 1 [18].

### 2.1. Clonal Selection

Clonal selection represents the classic Darwinian Model of residual lesions, where tolerant or weakly tolerant clones that existed before treatment are selected and amplified under drug pressure. Treatment stress acts like environmental selection, preferentially eliminating treatment-sensitive tumor cell clones while allowing pre-existing drug-resistant clones to survive and eventually proliferate, becoming the basis for tumor recurrence [45]. Recent study further shows that under drug stress, different subclones can exhibit highly repetitive and predictable selection trajectories, suggesting that drug resistance does not occur randomly but depends on initial clonal structure and innate fitness differences [56].

Within this framework, some cells in the residual lesions do not need to acquire new driver mutations; instead, they survive directly due to their existing genetic makeup and high fitness. For NICT, this means that the “survivors” seen in postoperative residual tissue may not be newly developed resistance products during treatment, but rather clones that existed before treatment and were only revealed after treatment selection [15,57]. This explanation is significant for understanding incomplete pathological remission, postoperative relapse, and failed retreatment.

### 2.2. Adaptive Plasticity

In contrast, adaptive plasticity emphasizes that another type of residual cells is not stable drug resistance, but rather enters a reversible adaptive state under treatment stress [58]. These states include slow cycle, dormancy, stress tolerance, enhanced stemness, hybrid EMT, and low immune visibility, etc. [51,52] Their common feature is that cells temporarily reduce proliferation, enhance survival, and can return to a more primitive transcriptional state after the stress is relieved. Emerging evidence suggests that this adaptation is not entirely dependent on novel mutations, but is more likely driven by epigenetic reprogramming and switching of transcription factor networks [56].

Treatment stress, microenvironmental signals, and intrinsic epigenetic fluctuations can collectively induce tumor cells to switch between multiple states [52]; drug resistance can also be explained by a “one-to-many (epi)genotype-to-phenotype” mapping, meaning that the same genetic background can output multiple transcriptional states in different environments, a capability supported by stable epigenetic memory [56]. Therefore, the resistance observed in residual lesions does not necessarily signify real genetic immobilization, but may simply be a reversible, plastic adaptation that can rebound after treatment. This is why residual tumors often recur after initial shrinkage: they are not simply survived, but rather learned to survive under stress.

### 2.3. Ecological Protection

However, even with genetic selection advantages or epigenetic plasticity, residual tumor cells still require an environment that allows them to survive, this is called ecological protection. The survival of residual tumor cells relies not only on intrinsic cellular properties but also heavily on protective niches within the tumor microenvironment (TME) [59,60]. Cancer-associated fibroblasts (CAFs), tumor-associated macrophages (TAMs), myeloid-derived suppressor cells (MDSCs), regulatory T cells (Tregs), aberrant vasculature, hypoxia, extracellular matrix (ECM) barriers and metabolic stress jointly construct such protective niches for residual tumor cells [61].

CAFs can promote tumor cell migration, stemness, and treatment resistance through ECM remodeling, paracrine factors, and mechanotransmission; while immunosuppressive cell populations reduce immune clearance efficiency by weakening effector T cell activity, allowing residual cells to hide or remain in a low-responsive state [62,63]. Hypoxia and abnormal blood supply not only limit drug penetration but also drive EMT, metabolic reprogramming, and stress survival through hypoxia-inducible factor (HIF)-related processes. For residual lesions after NICT, the TME is not a passive background but an active “third-party force” involved in selection and protection: it both selects cells that can adapt to hypoxia, low nutrition, and immune stress, and helps these cells maintain reversible tolerance and recurrence potential [62,63,64].

**Figure 2:**
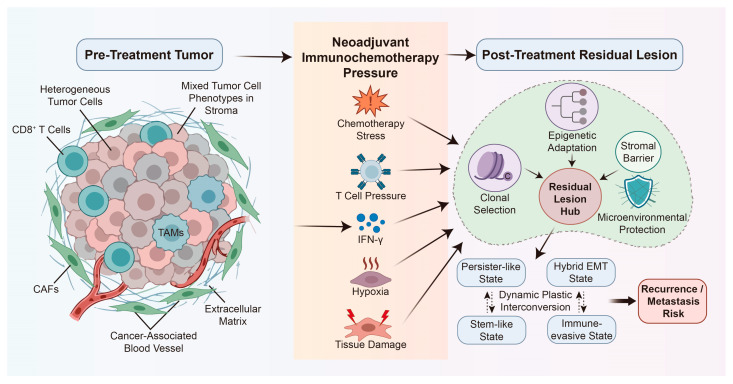
Non-apoptotic residual tumor cells within a therapy-shaped plasticity niche after NICT. The left side depicts the pre-treatment tumor ecosystem: composed of polyclonal heterogeneous tumor cells, CD8^+^ T cells, tumor-associated macrophages (TAMs), cancer-associated fibroblasts (CAFs), aberrant blood vessels, and fibrotic matrix. The middle section shows the convergence of multiple pressures exerted by NICT to the right, including chemotherapy stress, T-cell cytotoxic pressure, persistent interferon (IFN)-γ signaling, hypoxia, and tissue damage/wound-healing cues. The right side illustrates a residual plasticity niche in which pre-existing and inducible plastic states are selected, amplified, and stabilized by therapy-induced pressures, driven by three synergistic mechanisms: clonal selection, epigenetic adaptation, and microenvironmental protection, resulting in four dominant residual states: persistent-like/dormant, hybrid EMT/invasive-plastic, stem-like/regenerative, and immune-evasive. Bidirectional connections between these states indicate reversible transitions, collectively increasing recurrence/metastasis risk.

Therefore, the formation of residual lesions is not the result of a single clone or a single pathway, but rather the product of long-term co-evolution between the tumor cells themselves, their epigenetic state, and the surrounding niche (Figure 2).

## 3. Epigenetic Programs Underlying Residual Malignant Cell States

The four residual states described below:—persister-like/dormant, hybrid EMT/invasive plastic, stem-like/regenerative, and immune-evasive—are intended as analytical categories, not as rigid, mutually exclusive cell types. In reality, these states represent probabilistic attractors on a deformable epigenetic landscape: cells can occupy intermediate positions, co-express features of multiple states, and transition between states under changing selection pressures [65]. A pan-cancer single-cell analysis across 15 tumor types identified six gene modules whose expression defines recurrent cancer cell states, including stress response, EMT, and interferon response that constitute general features of cancer rather than properties of individual tumor types [66]. This convergence supports the cross-tumor-type applicability of the states described here. However, the precise molecular implementation of each state is constrained by the developmental origin of the tumor: a developmental constraint model proposes that cancer cells can only access states that are developmentally reachable from their cell of origin, which explains why, for example, EMT programs differ between epithelial and mesenchymal cancers, and why stem-like programs in lung adenocarcinoma recapitulate lung developmental hierarchies rather than intestinal or neural ones [67]. Furthermore, different genetic backgrounds can converge on the same catalog of states but alter their relative frequencies: a plastigenic process in which genetic alterations modulate state abundance rather than state identity [67]. Therefore, the four-state taxonomy should be interpreted as a working framework for organizing mechanistic insights, while recognizing that the boundaries between states are porous, the specific markers and regulators are tissue-context-dependent, and the dynamic interconversion between states is itself a biologically and clinically relevant property.

### 3.1. Persister-like and Dormant States

Post-treatment residual tumor cells often enter a DTP-like state or dormant state: these cells typically exhibit slow cell cycle/low proliferation, enhanced anti-apoptosis and stress tolerance, and transient, broad-spectrum, and reversible tolerance to multiple treatments [68,69]. When treatment is stopped or the microenvironmental stress changes, these cells can re-enter the proliferation program and drive relapse [70]. Therefore, this state can well explain the long-term existence and late relapse of minimal residual disease (MRD). Clinically, even after neoadjuvant therapy achieves complete pathological remission, residual tumor cells can still be detected in the resected specimen, suggesting that residual does not mean quiet and harmless, but rather resembles an adaptive reservoir temporarily suppressed by treatment [71,72].

At the epigenetic level, the stable maintenance of the pesrister/dormant state depends on reversible chromatin remodeling, rather than de novo mutations [72]. It is generally believed that these cells are often accompanied by enhanced repressive chromatin programs, including increased H3K27me3/H3K9me3, Polycomb-related silencing, chromatin compression, and widespread transcriptional silencing; moreover, histone deacetylase (HDAC)-mediated deacetylation is also considered one of the upstream mechanisms for maintaining low transcriptional activity [73,74,75].

Meanwhile, metabolic adaptation is essential for persister survival. DTP cells are not truly metabolic shut down, but maintain a minimum level of survival and subsequent resuscitation capacity through redox balance, lipid metabolism/oxidative phosphorylation (OXPHOS) reprogramming, and switching of other energy pathways [76,77]. Recent studies on residual disease in lung cancer further demonstrate that residual lesions can be maintained by specific signaling axes and synergize with persisters, indicating that “resting” itself is a highly organized adaptive program, rather than a simple proliferative halt [78]. Therefore, the persister-like/dormant state can be summarized as a reversible survival program shaped by epigenetic locking, transcriptional silencing, and metabolic resetting, and is one of the core relapse seeds of residual lesions.

### 3.2. Hybrid EMT and Invasive Plasticity

Hybrid EMT describes a cellular state in which cancer cells simultaneously co-express both epithelial and mesenchymal features, occupying a stable, functional equilibrium position along the epithelial–mesenchymal plasticity (EMP) landscape [79]. While “partial EMT” is sometimes used interchangeably, the two terms carry subtly different emphases: partial EMT underscores the incompleteness of the transition from an epithelial origin, whereas hybrid EMT emphasizes the functional co-existence of proliferative (epithelial) and invasive (mesenchymal) programs, a dual capacity that is uniquely advantageous for residual tumor cells (Table 1) [80]. Biologically, the hybrid state is not a default consequence of an interrupted transition but is actively maintained by core regulatory networks: the ZEB1/ZEB2–miR-200 and SNAIL1/SNAIL2–miR-34 double-negative feedback loops, coupled to epigenetic stabilizers such as H3K27ac remodeling at enhancers of EMT transcription factors [80,81]. EMP enables cells to traverse the epithelial–mesenchymal landscape bidirectionally, and the hybrid state represents the most fitness-optimized configuration for cells that must simultaneously preserve proliferative potential and acquire invasive, stem-like, and drug-tolerant properties [82,83,84,85]. The concept of EMT as a spectrum of intermediate states (rather than a binary switch) was first systematically articulated by Nieto et al., experimentally validated in vivo by Pastushenko et al. who identified multiple EMT subpopulations with distinct functional properties in skin cancer and mammary tumor models, and subsequently refined through consensus nomenclature [81,86]. Recent comprehensive reviews explicitly connect hybrid EMT to MRD, drug tolerance, and tumor dormancy, linking the concept directly to the themes of residual tumor plasticity [87,88,89,90].

Unlike the classic binary narrative of decreased E-cadherin and increased N-cadherin, residual tumors more often remain in a hybrid/partial EMT lineage: cells are not fully mesenchymalized, but retain some epithelial adhesion and proliferative capacity, and acquire migration, invasion, anti-apoptosis, and treatment resistance [97,98,99]. This mixed state is commonly found at the invasive front, tumor buds, and circulating tumor cell (CTC) clusters, and is associated with stronger tumor initiation, dissemination, and poor prognosis in various cancer types; in other words, the most dangerous aspect of residual cells is not complete EMT, but this reversible and switchable transitional state.

Mechanistically, hybrid EMT resembles enhancer-switching-driven transcriptional reprogramming rather than a simple flipping of a single marker [97]. Enhancers associated with invasion, stemness and stress adaptation acquire H3K27ac and establish a more open chromatin landscape [97]. AP-1, TEAD and ZEB1/SNAIL/TWIST collectively serve as core hubs for state transition [100]. These transcription factors do not function uniformly across all cancer types. Instead, they determine whether cells remain in an epithelial migratory state or progress toward a highly invasive mesenchymal program, contingent on specific tissue context and therapeutic stress. Notably, the YAP/TAZ-TEAD axis merits particular emphasis, which is tightly coupled with EMT, cancer stemness and invasiveness, and its dependency signature can predict TEAD dependence across platforms in multiple cancer types, suggesting this axis acts as a critical hub driving residual cells into an invasive plastic state [101,102].

Functionally, hybrid EMT often occurs concurrently with immune rejection and dissemination dominance. Studies in pancreatic cancer show that EMT-related transcription factors ZEB1 and SNAIL act as dual genetic and epigenetic regulators, driving Irf6 silencing and thus weakening tumor cell sensitivity to T cell killing and TNF-α-induced apoptosis [103]. In a neuroendocrine transdifferentiation model of prostate cancer, the CXCR4-LASP1-G9a-SNAIL axis further demonstrates that EMT is not a static marker change, but a state reconnection process amplified by both the microenvironment and epigenetics [104]. Also, background alterations such as p53 and LKB1 can also release the brakes on plasticity, making EMT/partial EMT more likely to coexist with chromatin instability, transdifferentiation, and invasiveness [105,106]. Therefore, in residual lesions after NICT, hybrid EMT/invasive plasticity is more suitable as a second-layer survival strategy: it enhances invasion and immune escape while preserving proliferative potential, pushing a few surviving cells into a transitional state most conducive to recurrence.

### 3.3. Stem-like and Regenerative States

Post-treatment tissue damage and inflammation repair can push some residual tumor cells into a stem-like/regenerative-like state. This state does not mean the existence of a fixed, static subset of cancer stem cells within the tumor; more accurately, it is a dynamic cellular state that can be induced by treatment stress and microenvironmental signals [107,108]. In this state, cells maintain a certain level of survival and proliferation capacity while acquiring stronger self-renewal, clonal reconstitution, and reversible differentiation, thus making them more likely to re-expand and participate in recurrence after treatment withdrawal. In other words, the stemness in residual lesions is more like a functional program under stress than a pre-set lineage endpoint. Mechanistically, this process is often reactivated by developmental signals such as Wnt, Notch, and Hedgehog, and works synergistically with core transcription factors such as SOX2, KLF4, and MYC [109]. Together, they propel residual cells back to a low-differentiation, highly plastic, and regenerative transcriptional state. Among these, YAP/TAZ, as the hub of mechanical and regenerative signals, is often located upstream in this network and functionally coupled with SOX2, supporting spheroidization, metabolic adaptation, and treatment tolerance [108,110]. In different tumor contexts, YAP/TAZ can also continuously amplify the regenerative-like transcriptional program through crosstalk with Notch, Wnt/β-catenin, and Hedgehog, enabling residual cells to retain certain epithelial characteristics while acquiring stronger regenerative and invasive potential [111].

At the epigenetic level, stem-like states are often accompanied by super-enhancer remodeling. Therapeutic stress can rewrite the enhancer accessibility of key transcription factors, reactivating a few core gene clusters and forming stable state memories. Recent studies have shown that SOX2 can inhibit or rearrange MYC transcription by altering the coactivator composition of the c-MYC super-enhancer; YAP/TAZ are also frequently incorporated into this enhancer-driven network, participating in maintaining stemness and regeneration programs [112,113]. Therefore, in residual lesions after NICT, the stem-like/regenerative-like state can be summarized as: therapeutic damage → regeneration signal activation → YAP/TAZ-Notch/Wnt/Hedgehog axis initiation → super-enhancer remodeling → stemness program reconstruction. This state is beneficial for short-term survival and provides the most plastic cellular reserve for subsequent relapse [107,112,114].

### 3.4. Immune-Evasive States

Another key survival strategy for residual tumor cells is to enter an immune-evasive/immune-low-visible state. This state is most directly related to immunotherapy, and its core characteristic is not simply escaping killing, but rather reducing the probability of being recognized: antigen processing and presentation capabilities decrease, MHC-I, B2M, and TAP1/TAP2-related pathways weaken, IFN-γ response is reprogrammed, and immunosuppressive molecules such as PD-L1 and CD47 are upregulated, ultimately creating a microenvironment unfriendly to CD8^+^ T cells and phagocytes [115,116,117]. In clinical samples, residual GC after anti-PD-1 therapy shows downregulation of HLA class I/MHC class II in tumor cells, suggesting that immune escape is not necessarily complete disappearance, but may be adaptive survival after reduced antigen visibility [118].

It is important to emphasize that this type of immune escape is not always driven by irreversible gene mutations. While abnormalities in pathways such as B2M, JAK1, and NLRC5 can indeed lead to stable antigen presentation defects or IFN-γ insensitivity, increasing evidence suggests that the low visibility of immunity in residual lesions is often maintained by chromatin state, transcription factor networks, and metabolic programs, and therefore has a degree of reversibility [119,120,121]. For example, epigenetic drugs can restore the expression of antigen presentation machinery such as MHC-I, B2M, PSMB8/9, and TAP1/TAP2, indicating that “turning off the presentation program” itself can be turned back on; however, this may also be accompanied by PD-L1 upregulation, suggesting that residual cells will rebalance between restoring antigenicity and maintaining inhibitory activity [122]. Similarly, IFN-γ may enhance anti-tumor immunity or induce tumor cells to remodel PD-L1, YTHDF2, or EMT-related programs, thereby converting inflammatory signals into an escape advantage [121,123].

Furthermore, immune escape is often closely coupled with EMT/hybrid EMT. In PD-L1-high NSCLC, the higher the EMT signature, the more likely there is a decrease in T cell infiltration, an increase in Treg and M2 macrophages, and a poorer response of immune checkpoint inhibitor (ICI); while EMT transcription factors such as TWIST1 and ZEB1 can directly promote PD-L1 expression, further amplifying the immune rejection effect [123,124]. This indicates that residual cells do not simply hide, but rather rewrite their immune interfaces simultaneously at the transcriptional and epigenetic levels, making them both more difficult to identify and more likely to push local TME into a cold tumor state [125,126].

Therefore, the immune-evasive state can be summarized as: weakened antigen presentation + IFN-γ pathway reprogramming + upregulation of immunosuppressive molecules + chemokine/EMT-driven T cell rejection. This state directly explains why some lesions, despite appearing to shrink after treatment, can still survive long-term under immune pressure and subsequently become a source of relapse (Table 2) [115,116,125]. Table 2 serves as a mechanistic reference summarizing the biological features of each residual cell state as characterized in post-treatment surgical specimens; it is not intended as a pre-treatment diagnostic panel. The translational counterpart: mapping each state to actionable therapeutic strategies will provide in Section 5.3.

## 4. Microenvironmental Niches Reinforcing Residual Plasticity

### 4.1. Immune Pressure and Immune Editing

Following neoadjuvant immunotherapy, residual tumor cells are not a passive group that luckily survives, but rather an adaptive subset that is reselected under continuous immune pressure. Cells easily recognized and killed by T cells are eliminated first, while those remaining are often cells with weaker antigen presentation, impaired IFN-γ responses, or those more prone to forming immune rejection phenotypes [115,127,128]. Therefore, the role of immunotherapy is not merely killing tumors, but also directly shaping the state of residual cells through immune editing, causing them to evolve towards lower visibility, lower immunogenicity, and greater plasticity.

The key to this process is not necessarily new irreversible gene mutations, but often the selection and reprogramming of existing cellular states. Classic alterations include weakening of the MHC-I/B2M/TAP1-TAP2-related antigen processing and presentation pathways, inactivation of the IFN-γ-dependent JAK/STAT axis, or epigenetic silencing of antigen presentation machinery, thereby reducing the ability of tumor cells to recognize CD8^+^ T cells [129,130]. Recent studies have further suggested that antigen visibility in residual tumors is not a static property, but rather changes dynamically under treatment stress; some cells can simultaneously carry B2M deficiency and IFN signaling defects, forming a more thorough T cell escape phenotype [127,131].

Meanwhile, immune pressure itself also reshapes the transcriptional state of tumor cells. Sustained IFN-γ exposure can enhance antigen presentation and inflammatory responses, and may also induce PD-L1 and LAG-3-related adaptive feedback, even pushing cells into a less differentiated and more tolerant state [132,133]. This means that while the immune system is clearing tumors, it is also “training” surviving cells to learn how to survive. In other words, immune pressure is not a one-way elimination mechanism, but a dual process of selection and state shaping: it determines both who is eliminated and in what state those remain [134,135].

Therefore, immune escape is not always a terminal event, but rather a plastic state formed under treatment stress. The reason why residual cells can persist is often not because they have completely lost their immune-related functions, but because they have transformed the immune system’s attack into a signal for self-state reprogramming, thereby gaining stronger long-term survival ability and subsequent relapse potential.

### 4.2. Myeloid-Rich Wound-Healing Niches

While chemotherapy and immunotherapy kill tumor cells, they also cause tissue damage, release of necrotic debris, and local inflammation, triggering a wound-healing-like myeloid response [136,137]. During this process, TAMs and MDSCs are often recruited and enriched, accompanied by the upregulation of repair/inhibitory molecules such as IL-10, TGF-β, ARG1, SPP1, and VEGF [137,138]. These signals promote angiogenesis, matrix remodeling, and fibrosis repair, while simultaneously suppressing effector T cell activity, thus transforming the process originally intended to clear damage into a barrier protecting residual tumor cells.

In fibrosis-associated hepatocellular carcinoma (HCC), studies have shown that TGF-β can drive monocyte differentiation into MDSCs and enhance immunosuppression and reduce T cell proliferation through the PPP1R15A-ARG1/S100A8/9 axis; inhibiting this axis can reduce M-MDSCs and enhance the efficacy of ICI therapy [138]. This suggests that the repair response activated after treatment is not necessarily a passive process of host self-recovery, but may be actively utilized by residual tumor cells, gradually transforming into an immune-protective niche. Therefore, this type of myeloid-rich wound-healing niche is essentially a repair-suppression complex state dominated by myeloid cells, providing a niche basis for the continued survival of residual tumors and subsequent recurrence [136].

### 4.3. CAF-ECM-Fibrotic Niches

CAF and ECM constitute the most typical physical-signal dual barrier in residual lesions. After treatment, ECM-producing myCAF/ECM-myCAF are often retained or remodeled in the residual area, promoting tumor cell survival and restricting CD8^+^ T cell entry through collagen deposition, matrix stiffening (ECM stiffening), and YAP1/TEAD activation [139,140]. In ovarian cancer, residual ANTXR1^+^ myofibroblasts after chemotherapy can suppress anti-tumor immunity through a YAP1-dependent mechanism; in breast cancer, ECM-myCAF/wound-myCAF/TGFβ-myCAF spatially co-localize with high TGF-β signaling, while ECM regulators can alter local TGF-β activity and affect CD8^+^ T cell infiltration [139,141]. These fibrotic niches are also often accompanied by enhanced chemokine programs (such as CXCL12) and LRRC15-related matrix programs, thereby further strengthening immune rejection, promoting hybrid EMT, and improving treatment tolerance [142,143]. Therefore, the CAF-ECM-fibrotic niche is not simply a scar-like background, but an immune isolation zone established by residual tumors using tissue repair processes, which can sustainably support survival and proliferation before recurrence [144].

### 4.4. Hypoxic and Metabolic Niches

Residual tumor regions are typically accompanied by abnormal blood vessels, persistent hypoxia, lactate accumulation, adenosine production, and nutrient competition, creating a local microenvironment characterized by hypoxic and metabolic stress. In these niches, HIF signaling, the AMPK-mTOR axis, and mitochondrial metabolic reprogramming work together to shift cells from a “proliferation-first” to a “survival-first” approach, further enhancing the plasticity and tolerance of residual cells [145,146]. More importantly, these metabolic changes are not merely energy adaptations but directly alter the supply of epigenetic substrates: NAD^+^ influences redox and deacetylation processes, acetyl-CoA determines histone acetylation levels, and α-KG and SAM connect demethylation and methylation reactions, respectively, thereby translating metabolic state into chromatin state [147,148].

Under hypoxic conditions, tumor cells often induce glycolysis, inhibit mitochondrial oxidation, and increase lactate production through HIF-1α [149,150]. Lactate is not only a metabolic waste product but also a signaling molecule and epigenetic regulator, promoting cell cycle maintenance, invasion programs, and treatment tolerance. Recent studies suggest that lactate accumulation can also participate in tumor state remodeling through histone lactylation, enabling cells to maintain higher transcriptional heterogeneity and reversibility in hypoxic and low-nutrient environments [150,151]. Meanwhile, hypoxia also affects NAD metabolism and residual mitochondrial function; the Complex I activity retained under hypoxia can maintain NAD^+^ regeneration and support α-KG-related metabolism, thereby influencing cell fate and adaptability [147,152].

**Figure 3:**
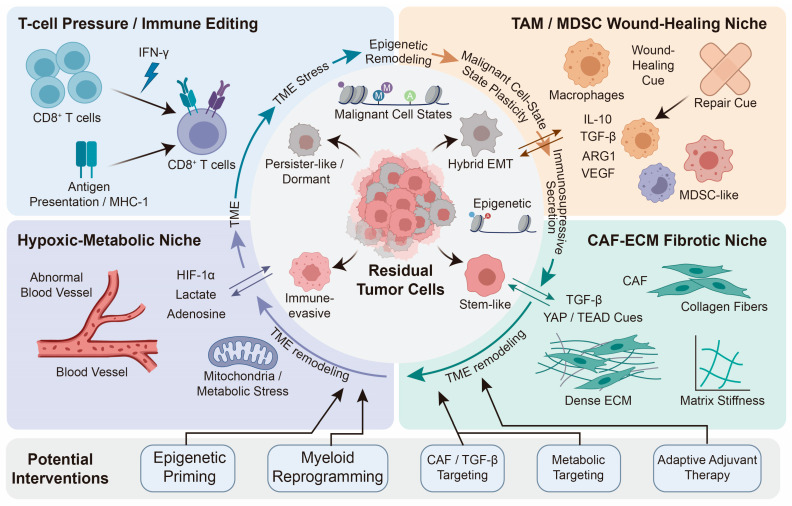
Reciprocal feedback between epigenetically plastic tumor cells and adaptive microenvironmental niches. In the middle: Residual tumor cells are labeled with four plasticity states: persister-like/dormant, hybrid EMT, stem-like, and immune-evasive. Peripheral niches: T cell stress/immune editing, TAM/MDSC wound healing niche, CAF-ECM fibrosis niche, and hypoxia-metabolic niche. Bidirectional arrows indicate a closed loop: TME stress → epigenetic remodeling → malignant cell state plasticity → immunosuppressive secretion group → TME remodeling. The bottom lists potential interventions: epigenetic priming, myeloid reprogramming, CAF/TGF-β targeting, metabolic targeting, and adaptive adjuvant therapy.

Therefore, the key significance of the hypoxic and metabolic niche lies not in hypoxia itself, but in its transformation of microenvironmental stress into an inheritable epigenetic program. Residual tumor cells, through this program, can maintain a stem-like, persister-like, or invasive state under hypoxic, hyponutrient, and high lactate conditions, and proliferate again after the therapeutic stress is relieved [146,153]. This is precisely the logic this review aims to emphasize: metabolic stress converts microenvironmental adaptation into epigenetic plasticity (Figure 3).

### 4.5. Apoptosis-Induced Repopulation Signaling: Therapy as a Niche Sculptor

An underappreciated dimension of residual niche biology is that cytotoxic therapy itself directly generates pro-regenerative signals that promote the survival and expansion of residual tumor cells. This phenomenon, called “Phoenix Rising”, was first described by Huang et al., who demonstrated that caspase-3 activation in dying tumor cells triggers the iPLA2–COX-2–PGE2 axis, leading to STAT3-dependent proliferation of surviving tumor cells [49,154]. Importantly, higher levels of activated caspase-3 in tumor tissues correlated with significantly increased rates of recurrence and death in patients, providing direct clinical evidence for the relevance of this pathway. Kurtova et al. subsequently showed that Prostaglandin (PG) E2 released from chemotherapy-killed bladder cancer cells stimulates cancer stem cell proliferation and drives tumor repopulation between treatment cycles; pharmacological blockade of this axis with the cyclooxygenase-2 (COX-2). COX-2 inhibitor celecoxib abrogated chemoresistance [155].

**Figure 4:**
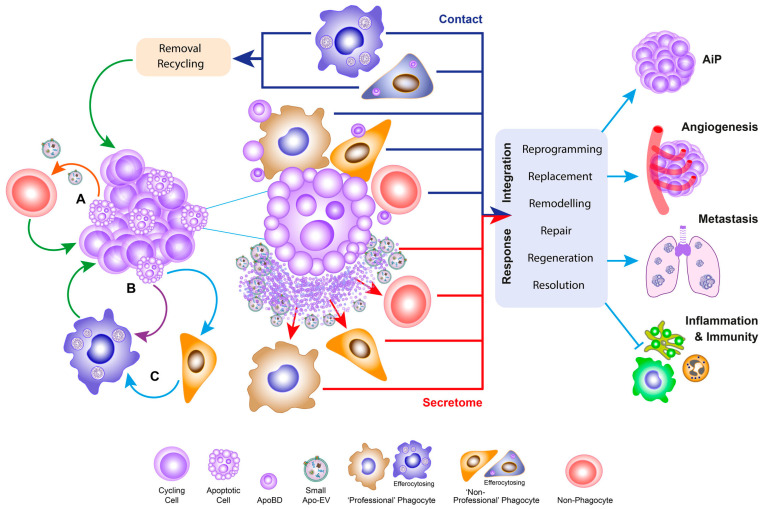
Overview of the potential oncogenic effects of apoptosis in tumor tissues. Representation of intercellular signals from apoptotic cells in a growing tumor, which are mediated either through the apoptotic secretome, including small apoptotic extracellular vesicle (ApoEV) production (lower center, red pathways), or via contact-dependent means (upper center, dark blue pathways). Response signals are integrated into pro-oncogenic effector programs (right) such as trophic apoptosis-induced proliferation (AiP), angiogenesis, invasion, enhanced migration and metastasis, resolution and anti-inflammatory signaling as well as suppression of pro-inflammatory effects and of innate and adaptive anti-tumor immunity. Recycling of digested apoptotic cell-derived components (top left) also very likely helps support tumor growth. Macrophages probably play critical roles in many of these responses and comprise the major professional efferocytic cells of the TME and probably respond, independently of efferocytosis to contact-mediated and secretory communication from apoptotic cells too. Notably, virtually any cell in the malignant tissue has the potential to respond to the various signals emanating from apoptotic cells, some acting as professional efferocytes. In this way, it is proposed that intricate, intercellular circuits (left), founded in apoptosis, can be established to support disease development. Illustrative, theoretical examples of such circuits are shown in which the endpoint in each case is pro-oncogenic (green arrows). In circuit A (orange arrow), contactless stimulation of a non-phagocyte (e.g., lymphocyte) of the TME is shown. In B (purple arrow), the response is that of an efferocytosing macrophage, while C is more complex (blue arrows) depicting communication between non-professional and professional phagocytes. (Reproduced with permission from Ref. [162]. 2023 John Wiley & Sons Ltd.).

The apoptotic secretome extends well beyond PGE2, encompassing soluble mediators, damage-associated molecular patterns (DAMPs), cytokines/chemokines, lipids, and apoptotic extracellular vesicles/apoptotic bodies [156]. Dying cells can emit ATP and HMGB1, expose calreticulin on the cell surface, and present or release HSP-associated danger signals, which are sensed by receptors such as purinergic receptors, Toll-like receptors (TLRs), RAGE and CD91 to activate NF-κB-dependent inflammatory or reparative signaling in neighboring immune, stromal, and surviving tumor cells [157,158,159]. In parallel, apoptotic or therapy-damaged cells can promote compensatory proliferation and tumor repopulation through Wnt/Wingless-like mitogens, Hedgehog/Wnt pathway remodeling, ATP-driven macrophage amphiregulin production, and cytokine/chemokine-rich apoptotic bodies, thereby allowing tumors to hijack wound-healing-like repair programs after cytotoxic therapy [159,160,161]. The broader conceptual framework of apoptosis-driven remodeling of the TME is illustrated in Figure 4 of apoptosis-centered oncogenic signaling, in which apoptotic tumor cells communicate through soluble secretome components, apoptotic extracellular vesicles, and contact-dependent pathways to promote apoptosis-induced proliferation, angiogenesis, invasion, metastasis, reparative macrophage programs, and immune suppression [162].

In the NICT setting, this apoptosis-driven regeneration mechanism may be particularly relevant because both cytotoxic chemotherapy and immune-mediated tumor killing can increase the burden of dying tumor cells. Chemotherapy can induce apoptosis through DNA damage and DNA damage response pathways, whereas immune checkpoint blockade can enhance CD8^+^ T-cell cytotoxicity and tumor apoptosis, including granzyme/perforin-dependent killing and death receptor-associated apoptosis [163]. The resulting apoptotic debris and secretome may provide trophic and inflammatory cues to residual tumor cells. Preclinical studies show that dying tumor cells can stimulate tumor repopulation through caspase-3-dependent PGE2 production and through exosome-mediated support of tumor-repopulating cells [164]. Chemotherapy-induced COX-2/PGE2 signaling has also been shown to enhance cancer stemness, invasiveness, drug resistance, and repopulation in residual tumor cells [165]. Therefore, although direct clinical proof in NICT-treated residual lesions remains limited, the apoptotic secretome may contribute to the paradoxical emergence or selection of proliferative and stem-like residual tumor states after initial tumor shrinkage. This interpretation is consistent with evidence that cancer stem-like cells are enriched in post-treatment residual disease and with neoadjuvant immunotherapy datasets linking non-complete pathological response to higher residual tumor burden and stemness-associated features [166,167]. Targeting this apoptosis-driven regeneration loop: through COX-2/PGE2 inhibition, EP2/EP4 or EP4 antagonism, or selective modulation of DAMP pathways such as HMGB1 or ATP/P2 × 7R may represent a rational adjuvant strategy to limit post-NICT tumor repopulation [164,168,169]. However, because DAMPs can also support ICD and antitumor immunity, such approaches should be context- and timing-dependent rather than broad DAMP suppression.

## 5. Translational Implications: Assessing and Targeting the Residual State

### 5.1. From Residual Volume to Residual State

Traditional indicators such as pCR, MPR, TRG/TRS, ypTNM, and R0 resection have become the most commonly used efficacy endpoints after neoadjuvant therapy [170,171,172]. However, they essentially still primarily answer the question of “how much is left”, i.e., the residual tumor burden, rather than “what state the remaining tumor is in”. In resectable NSCLC, the need for standardized sampling and stratified interpretation in postoperative pathological evaluation has been emphasized, because residual lesions exhibit significant spatial heterogeneity [173]. In other words, simply summarizing the entire residual lesion based on the proportion of viable tumor is often insufficient to reflect its true biological consequences.

Therefore, assessments of neoadjuvant therapy should evolve from residual volume to residual-state profiling: in addition to residual quantity, it should also determine whether residual cells are persister-like, whether they retain hybrid EMT/stem-like programs, whether antigen presentation and IFN responses have been reprogrammed, and whether they are embedded in TAM-rich, CAF-rich, hypoxic, or immune-excluded niches [70,144,174]. What truly determines relapse potential is often not simply residual volume, but rather the type of plasticity program and microenvironment niche that sustains the residual cells.

From a translational perspective, circulating tumor DNA (ctDNA), molecular subtyping, and spatial pathology should be complementary rather than substitutive. ctDNA can indicate recurrence risk, microscopic residual mapping can improve recurrence prediction, and spatial omics can further reveal the cellular state and tissue structure of residual lesions [175,176,177]. Therefore, the ideal postoperative assessment framework in the future should include both residual volume and residual state, and determine subsequent adjuvant therapy, de-enhancing, or microenvironment reprogramming strategies accordingly [78,178]. While these individual modalities are already established, the framework’s translational contribution lies not in the tools themselves but in their integrated purpose: shifting from binary risk stratification to state-guided therapeutic selection, whereby the specific residual cell state directs plasticity-locking strategies that have no counterpart in current burden-based adjuvant decision-making, which is discussed in Section 5.3.

### 5.2. Technologies for Residual-State Profiling

The core value of these technologies lies not in their individual “more advanced” aspects, but in their shared ability to answer the same translational question: In what state are the residual tumor cells, and which microenvironmental niche protects them? Paired pre-treatment biopsies and post-treatment/residual lesion specimens provide the most basic timeline anchors, allowing us to directly compare the state transitions of the same patient before and after treatment; while single-cell RNA sequencing (scRNA-seq) further breaks down residual lesions from quantity into different cellular states, such as persister-like, stem-like, hybrid EMT-like, or immune-evasive programs [179,180]. Building upon this, scATAC-seq and single-cell multiome are significant because they connect state changes to chromatin accessibility and transcription factor networks, thus explaining why these residual states can be maintained, switched, or reprogrammed [181,182]. Spatial transcriptomics and multiplex immunohistochemistry (IHC) & immunofluorescence (IF) answer another crucial question: where do these states occur, and are they enclosed in TAM-rich, CAF-rich, hypoxic, or immune-excluded niches? Recently, in glioma and HCC residual disease, spatial omics has directly revealed the spatial coupling between myeloid cells, hypoxia, stem-like tumor cells, and CD8 exhaustion, suggesting that residual states are essentially a spatial phenomenon shaped by the microenvironment [180,183,184].

While ctDNA/MRD monitoring does not directly define cell state, it provides the most practical longitudinal readout: persistent ctDNA positivity post-surgery often indicates the presence of amplifiable residual clones, the risk of recurrence of which has been validated in both NSCLC and colon cancer [185,186]. Organoid-immune co-culture provides a causal validation platform for this framework: we can place patient-derived residual cells under specific immune stress, myeloid signaling, or metabolic conditions to test whether they maintain a persister state and identify reversible nodes [180,187]. Therefore, the closed loop of residual-state profiling should be “state definition—niche location—longitudinal tracking—functional validation”.

### 5.3. Residual-State-Guided Therapeutic Strategies

To sum up, it has a sense of concept that a “plasticity-locking therapy” strategy could be put forward. Its goal is not simply to continue killing more, but to prevent residual cells from escaping into a persister-like, stem-like, or immune-evasive state, locking residual lesions within a window where they are easier to eliminate, or at least less likely to recur [70,144].

First, epigenetic priming. Residual cells often reduce antigen visibility, weaken IFN responses, and gain greater plasticity through epigenetic reprogramming; these changes are particularly crucial in dormancy and therapy-persistent residual disease [70]. For example, HER2-low triple negative breast cancer shows HLA-related site hypermethylation accompanied by immune escape, while residual breast cancer cells can enter a non-hereditary dormancy/awakening state after long-term endocrine therapy [75,188]. Therefore, for residual lesions with low antigen-presentation, a “warm-up” strategy using related approaches of DNMT/HDAC/EZH2/BET/LSD1/CBP-p300 can be considered before ICI to restore IFN-driven immunogenicity and antigen presentation [189,190,191,192,193,194].

Second, niche targeting. Residual tumors do not survive solely through intracellular processes, they also depend on a protective niche comprising CAF, TAM, MDSC, TGF-β, VEGF, and metabolic immunosuppression. CAF-mediated rescue and vasculature-limited drug delivery are two major spatial mechanisms of residual disease, while fibrosis and the TGF-β axis can directly shape immune rejection [70,144]. Therefore, if residual lesions are CAF/TGFβ-high, anti-TGF-β or CAF-targeted combination therapy should be prioritized; if they are TAM/MDSC-rich, myeloid remodeling (such as CSF1R, CCR2/CXCR2, or adenosine pathway intervention) is more suitable.

Third, adaptive adjuvant therapy. Postoperative adjuvant therapy should not be stratified solely by ypTNM and TRG/TRS, but should incorporate a closed-loop decision-making process based on residual-state profiling and ctDNA/MRD. Longitudinally negative patients do not necessarily require intensive adjuvant therapy, and dynamic monitoring of MRD can support an adaptive framework of “monitoring–intervention–remonitoring”. Based on this, state-oriented postoperative strategies can be formed: antigen-presentation-low → epigenetic priming + ICI; CAF/TGFβ-high → anti-TGFβ/CAF targeting; TAM/MDSC-rich → myeloid reprogramming; IFN-high but exhausted → non-PD-1 checkpoints or co-stimulatory targets. The unit of future adjuvant therapy will no longer be just tumor burden, but residual state (Table 3).

### 5.4. Targeting State-Specific Therapeutic Vulnerabilities

Beyond the broad strategies of epigenetic priming and niche disruption discussed above, emerging evidence reveals that DTP cells acquire specific dependencies that create exploitable therapeutic vulnerabilities: a concept central to synthetic lethality approaches against residual disease.

#### 5.4.1. Ferroptosis Vulnerability

The most validated DTP-specific vulnerability is their acquired dependency on glutathione peroxidase 4 (GPX4). Persister cells across multiple cancer types, including breast cancer, melanoma, lung cancer, and ovarian cancer, downregulate global antioxidant programs and become critically dependent on GPX4 to prevent lipid peroxidation and ferroptotic cell death [34]. GPX4 inhibition (e.g., RSL3, ML210) selectively kills persister cells while sparing drug-sensitive parental cells, and prevents tumor relapse in mouse models [34]. More recently, fentomycin-1, a lysosome-targeted compound that exploits the iron addiction of DTP cells, which upregulate CD44-mediated iron endocytosis, to catalyze lysosomal membrane lipid peroxidation and trigger ferroptosis selectively in CD44-high persister subpopulations, with validated efficacy in an immunocompetent breast cancer metastasis model [195]. These findings suggest that ferroptosis-inducing agents may represent a rational strategy to eradicate persister-like residual cells after NICT, particularly in patients with residual-state profiling indicating a dominant persister/dormant state.

#### 5.4.2. Metabolic Synthetic Lethality

DTP cells can undergo metabolic reprogramming toward mitochondrial respiration/OXPHOS and lipid metabolism, including FAO, thereby creating targetable metabolic dependencies. This has been demonstrated in BRAF/MEK inhibitor-tolerant melanoma persisters and EGFR-TKI-induced lung cancer persister cells [196,197,198,199]. In addition, mitochondrial translation and mitochondrial biogenesis represent vulnerabilities of OXPHOS-dependent therapy-resistant or stem-like cancer cell states, as shown by studies using mitochondrial-targeted antibiotics such as tigecycline or doxycycline [200,201]. Finally, the ISR, particularly ISR-ATF4 signaling and PERK-dependent survival programs, has been linked to drug-tolerant or quiescent residual cancer cell states, supporting ISR/PERK-eIF2α-ATF4 signaling as a therapeutically targetable dependency [202,203,204].

#### 5.4.3. DNA Damage Repair Synthetic Lethality

DTP cells exhibit elevated mutation rates and increased reliance on DNA damage tolerance pathways, creating synthetic lethality with ATR, CHK1, and CHK2 inhibitors. This approach exploits the replication stress inherent to the persister state [205,206,207,208].

#### 5.4.4. Anti-Apoptotic Dependency

Notably, DTPs and immunotherapy persister cells (IPCs) share decreased mitochondrial apoptosis sensitivity and exhibit cross-resistance: DTPs resist T-cell killing, while IPCs resist drugs [209]. BH3 mimetics (e.g., navitoclax, venetoclax) can target the anti-apoptotic dependencies shared by both persister types, restoring sensitivity to chemotherapy and T-cell-mediated killing [209,210]. This cross-resistance is directly relevant to NICT, where residual cells survive both drug and immune pressure simultaneously.

These persister-specific vulnerabilities complement the broader plasticity-locking framework by providing concrete, mechanism-based therapeutic targets for each residual state (Table 3).

## 6. Perspective and Future Directions

### 6.1. Residual Disease as a Dynamic Therapeutic State

Residual disease after neoadjuvant therapy should be viewed not only as incomplete tumor eradication, but as a clinically informative window into therapy-induced adaptation. Importantly, residual tumor plasticity should not be interpreted as a process generated solely by neoadjuvant therapy. Rather, neoadjuvant therapy exposes and reshapes a pre-existing heterogeneous and plastic tumor ecosystem, leaving behind cellular states that are more capable of persistence, immune escape, and later relapse. Residual lesions can persist as biologically distinct compartments that retain relapse competence and may already inform adjuvant treatment decisions [211,212,213]. In this sense, the post-neoadjuvant residual lesion is not a passive remnant of treatment failure, but an active transitional state in which the evolutionary direction of the disease can still be modified.

A second major implication is that residual tumor state is shaped by the combined effects of epigenetic reprogramming and microenvironmental protection. Dormant breast cancer cells can enter heritable non-genetic adaptive states through epigenetic rewiring, while pancreatic cancer can acquire EMT-associated immune resistance through plasticity programs that directly reduce sensitivity to T-cell killing [75,103]. In parallel, residual disease is often maintained within stromal, hypoxic, and immune-modulatory niches that stabilize survival programs and preserve the capacity for later outgrowth [213,214]. These observations support a model in which relapse is not explained by residual volume alone, but by the interaction between cell-intrinsic plasticity and niche-level protection.

The translational challenge, therefore, is to move from measuring how much tumor remains to defining what residual state remains. Conventional endpoints such as pCR, TRG, ypTNM, and R0 resection remain essential, but they are insufficient on their own to explain why patients with similar pathological burden can have very different recurrence risks [211,215]. Residual-state profiling, combined with ctDNA/MRD monitoring and spatial pathology, could enable more rational postoperative risk stratification and help match adjuvant therapy to the biology of the remaining disease rather than to tumor size alone [213,215].

### 6.2. Current Limitations of the Residual-State Framework

A limitation of this review is its deliberate focus on NICT. While this choice maximizes mechanistic depth and clinical timeliness, it means that plasticity mechanisms unique to other neoadjuvant methods (such as targeted therapy resistance in HER2-positive breast cancer or radiation-induced plasticity) are not covered in comparable detail. Extending the “residual-state profiling” and “plasticity-locking” frameworks to these contexts represents an important future direction.

Second, the generalizability of the four-state taxonomy across tumor types and its relationship to cell state dynamics remains limited. The framework was constructed primarily from evidence in NSCLC, breast cancer, GC, and melanoma, tumor types in which NICT and neoadjuvant ICI trials have generated the richest single-cell and multi-omic datasets. Whether the same four states, with the same epigenetic drivers and microenvironmental dependencies, apply equally to tumors with different developmental origins (e.g., glioblastoma, sarcoma, or renal cell carcinoma) remains insufficiently tested. Cells may be drifting constantly in and out of certain states, which is not a weakness of the framework but a biologically grounded property: in the epigenetic landscape model, cancer increases landscape entropy, enabling stochastic transitions between attractor states that would be rarely occupied in normal cells [65]. Non-genetic resistance mechanisms, including reversible state transitions, are now recognized as a major and targetable form of therapeutic resistance [216]. The four-state taxonomy should therefore be understood not as a static classification but as a snapshot of metastable attractors: the clinically relevant question is not only which state a residual cell occupies at a given moment, but how rapidly and in which direction it transitions under therapeutic pressure. Current single-cell snapshot data can identify states but cannot fully resolve transition kinetics; trajectory inference and lineage tracing in paired pre- and post-treatment samples will be needed to map the transition probabilities between states and to identify the transition-state cells that may be most therapeutically vulnerable [217]. Acknowledging this dynamism does not undermine the utility of the four-state framework; rather, it highlights that the goal of plasticity-locking strategies should be to constrain transition capacity, not merely to eliminate cells in a given state.

A critical appraisal of the residual-state framework must confront a sobering reality: none of the four proposed cell states has been prospectively validated as a treatment-guiding biomarker in any solid tumor. This stands in sharp contrast to the rapid clinical translation of ctDNA-based MRD assessment. ctDNA-based MRD detection has demonstrated extraordinary prognostic power across multiple solid tumor types [175,215,218]. A 2026 consensus statement by the Asian Thoracic Oncology Research Group (ATORG) further underscores the growing clinical acceptance of ctDNA MRD in early stage NSCLC management [219]. Also, the partial EMT/cell-state biomarker remains gap. In contrast, biomarkers based on cell state (e.g., partial EMT gene expression signatures, hybrid EMT immunostaining) remain confined to retrospective tissue studies. While multiple retrospective analyses have correlated partial EMT signatures or tumor budding with patient outcomes in pancreatic, breast, and colorectal cancers, no prospective interventional trial has ever used a partial EMT or cell-state biomarker to assign therapy [85,220]. The reasons include spatial heterogeneity, temporal dynamics, lack of standardization, and difficulties in accessibility, unlike ctDNA, which can be longitudinally monitored via blood, cell-state assessment requires tissue, limiting dynamic application [173]. Therefore, the residual-state framework should be framed cautiously as a conceptual research model instead of a ready-to-use clinical tool. Its merits lie in guiding mechanistic studies on residual lesion recurrence, uncovering druggable preclinical pathways, and inspiring biomarker research. Clinical adoption will rely on blood-accessible surrogates (CTC profiles, cell-free RNA signatures, or ctDNA-derived residual state readouts) instead of direct tissue cell-state testing [175,218,221]. Specifically, the state-defining features described throughout this review constitute concrete candidate biomarkers for each residual state: MHC-I/B2M/TAP expression for the immune-evasive state (Section 3.4), CD44-high expression or GPX4-dependency for the persister/dormant state (Section 5.4.1) [34], partial EMT gene expression scores for the hybrid EMT state [85,220], and TGFβ signaling activity or αSMA-positive CAF density for the niche-protected state (Section 5.3). Their clinical implementation would follow a two-step path: first, retrospective validation in archived post-NICT surgical specimens using standardized IHC panels or spatial transcriptomic profiling; second, development of blood-accessible surrogates, particularly CTC-based cell-state profiling, which can capture EMT phenotype, PD-L1 expression, and antigen-presentation markers from circulating tumor cells [222], to enable longitudinal, non-invasive residual-state monitoring in prospective biomarker-defined trials. The model warrants modest interpretive framing until these surrogates gain prospective validation.

A further limitation of this review is the imbalance between the NICT-centric scope and the predominantly non-NICT origin of the underlying mechanistic evidence [223]. For the persister-like/dormant, stem-like/regenerative, and hybrid EMT/invasive-plasticity states, most foundational mechanisms were established in chemotherapy-treated, radiotherapy-treated, or pre-clinical model systems. Direct single-cell or spatial multi-omic profiling of NICT residual lesions is now emerging but remains limited in patient numbers and tumor types [224,225,226]. Where direct NICT evidence is absent, we have relied on mechanistic extrapolation from analogous neoadjuvant or adjuvant contexts, which we have flagged where applicable [227]. Future studies that prospectively profile NICT residual lesions with paired pre-treatment biopsies, using single-cell, spatial, and epigenomic platforms, will be essential to confirm that the four-state plasticity framework derived from mixed-treatment evidence holds in NICT-specific contexts, identify NICT-specific residual cell states (e.g., TREM2^+^ macrophage-driven immune evasion, APOA1^+^ tumor cell niches), and enable rational design of plasticity-locking adjuvant strategies [25].

### 6.3. Future Directions: Toward Residual-State-Guided Precision Therapy

An important complement to this review’s focus on residual disease is the question of what mechanisms drive pCR after NICT. From a biological perspective, pCR can be understood as the mirror image of the residual immune-evasive state: where residual disease is characterized by antigen presentation loss, IFN-γ pathway reprogramming, and immunosuppressive niche protection, pCR is associated with neoantigen-specific T-cell clonal expansion, enhanced MHC-I/B2M/TAP-dependent antigen presentation, robust IFN-γ response activation, and immunostimulatory niche conversion, including tertiary lymphoid structure (TLS) formation and M1 macrophage polarization [7,223]. This symmetry reinforces the centrality of immune-evasive plasticity as a determinant of treatment failure, and suggests that strategies designed to re-sensitize residual cells may recapitulate, at least in part, the immune biology of spontaneous CR.

A further question of translational importance is whether the residual plasticity mechanisms described here operate equivalently in micrometastatic deposits and spatially discontinuous (skip) lesions. Existing evidence suggests that response to NICT can be discordant between the primary tumor and regional lymph node metastases: in resectable NSCLC, complete nodal clearance (ypN0) occurs more frequently than complete primary tumor clearance (ypT0), and primary tumor pCR does not guarantee nodal clearance [228]. This discordance likely reflects fundamental differences in the local immune microenvironment: metastatic lymph nodes, particularly N2 stations, often display a less immune-activated phenotype than the primary tumor. Whether the taxonomy of residual plasticity states proposed here applies equally to skip lesions, bone marrow-disseminated tumor cells, and organ-specific micrometastases remains a critical unanswered question. Addressing it will require neoadjuvant trials that incorporate multi-site tissue sampling, spatial transcriptomic profiling of discontinuous tumor foci, and disseminated tumor cells’ characterization; this is a research agenda that extends beyond the current scope of this review but represents an essential next step for translating plasticity-informed strategies to the metastatic and MRD settings [179,184].

The next generation of neoadjuvant and adjuvant strategies may need to not only shrink tumors, but also expose, classify, and therapeutically lock residual tumor states before they evolve into relapse.

## 7. Conclusions

In conclusion, while residual disease after systemic therapy is a long-recognized clinical problem, the plasticity of non-apoptotic residual tumor cells framework adds value by integrating clonal selection, adaptive plasticity, and ecological protection into a unified, NICT-specific state taxonomy that bridges mechanistic biology with actionable residual-state profiling. Importantly, this term does not imply that residual cells possess a novel form of plasticity generated de novo by treatment. Rather, it refers to the therapy-selected, therapy-amplified, and niche-stabilized manifestation of intrinsic tumor plasticity within viable residual malignant cells that have escaped apoptotic elimination. However, the clinical utility of residual cell-state biomarkers remains substantially less mature than ctDNA-based MRD assessment. At present, partial EMT, hybrid EMT, stem-like, dormant, and immune-evasive states should be interpreted as mechanistic and exploratory features rather than validated clinical predictors. Future studies should determine whether integrating tissue-based residual-state profiling with ctDNA/MRD, pathological response, and spatial immune context can improve recurrence prediction beyond existing tools. Therapeutic strategies aimed at reversing or locking plastic states remain investigational and should be tested in biomarker-defined, prospectively designed studies before being considered clinically actionable.

## Figures and Tables

**Table 1:** Comparison of hybrid EMT and complete EMT/partial EMT.

Item	Hybrid EMT (Hybrid/Intermediate State)	Partial EMT (Broad Definition)/Complete EMT	References
**Definition**	A well-defined intermediate epithelial–mesenchymal state in which cells simultaneously express epithelial and mesenchymal markers. Regulatory circuits (e.g., miR-200/ZEB) are maintained at intermediate co-expression levels.	Partial EMT: a broad term describing any incomplete EMT process. Complete EMT: cells that have reached or closely resemble a fully mesenchymal phenotype.	[84,91]
**Molecular markers**	Retained E-cadherin, increased Vimentin, intermediate miR-200 and ZEB expression; frequently associated with H3K27ac-mediated chromatin remodeling.	Complete EMT: marked downregulation of E-cadherin and high Vimentin expression. Partial EMT (broad usage): marker combinations are variable and not standardized.	[79,92]
**Stability**	Can be stabilized by phenotypic stability factors (PSFs) such as OVOL and GRHL2; capable of existing as a stable state while retaining phenotypic plasticity and rapid switching ability.	Complete EMT is generally more stably locked into a mesenchymal phenotype. Partial EMT is often transient and represents an intermediate transition state.	[91,93]
**Functional consequences**	Promotes collective migration, circulating tumor cell (CTC) clusters, enhanced stemness, increased therapy resistance, and greater invasive potential.	Complete EMT favors single-cell migration. Partial EMT (broad usage) is associated with diverse functional phenotypes depending on the degree of transition.	[84,94]
**Detection methods**	Single-cell transcriptomics, EMT scoring, combined immunohistochemistry (E-cadherin + Vimentin), and spatial omics for identifying invasive fronts and tumor budding.	Conventional pathology or imaging cannot reliably distinguish these states; molecular profiling and single-cell analyses are generally required for accurate characterization.	[95,96]

**Table 2:** Epigenetic programs underlying residual malignant cell states after therapy.

Residual Malignant Cell State	Core Phenotype	Dominant Epigenetic Program	Key Regulators/Pathways	Biological Consequence	Therapeutic Implication
**Persister-like and dormant state**	Slow-cycling or quiescent cells; enhanced stress tolerance; reversible broad drug tolerance	Chromatin compaction, transcriptional silencing, increased repressive histone marks such as H3K9me3 and H3K27me3, HDAC-dependent deacetylation	HDACs, EZH2/EZH1, KDM5/KDM6, Polycomb programs, metabolic rewiring linked to OXPHOS, redox balance, and autophagy	Maintains MRD and enables late relapse after treatment withdrawal or microenvironmental change	Target chromatin repression, dormancy exit, and survival metabolism; combine epigenetic therapy with cytotoxic or targeted agents
**Hybrid EMT and invasive plasticity**	Partial EMT/mixed epithelial–mesenchymal state; preserved proliferation with increased migration, invasion, and anti-apoptotic capacity	Enhancer switching, increased H3K27ac at invasion- and plasticity-associated loci, transcriptional rewiring rather than full lineage conversion	YAP/TAZ-TEAD, AP-1, ZEB1, SNAIL, TWIST, Wnt/Notch crosstalk	Promotes invasion, dissemination, therapy tolerance, and immune exclusion while retaining enough epithelial traits for continued growth	Block plasticity circuits, super-enhancer activity, and YAP/TAZ-driven invasive programs
**Stem-like and regenerative state**	Self-renewal, clonogenic expansion, and reversible dedifferentiation; often induced by injury, inflammation, and tissue repair signals	Super-enhancer remodeling, activation of developmental/transcriptional programs, open chromatin at stemness loci	Wnt, Notch, Hedgehog, SOX2, KLF4, MYC, YAP/TAZ	Re-establishes a regenerative-like program that fuels clonal repopulation and recurrence	Target regeneration-associated transcriptional circuits and enhancer dependencies; prevent reactivation of stemness programs
**Immune-evasive state**	Reduced antigen visibility, impaired immune recognition, immune-suppressive microenvironment	Epigenetic repression of antigen presentation and IFN-response programs; chromatin-mediated rewiring of immune signaling	MHC-I/B2M/TAP1/TAP2 downregulation, IFN-γ response rewiring, PD-L1, CD47, GAL9, chemokine remodeling	Enables escape from cytotoxic T cells and myeloid clearance; supports immune escape and persistence under immunotherapy	Restore antigen presentation, reverse immune silencing, and combine epigenetic drugs with immunotherapy

**Table 3:** Residual-state-guided plasticity-locking therapeutic strategies after neoadjuvant therapy.

Strategy Category	Target	Core Logic	Applicable Residual State
**Epigenetic priming**	DNMT, HDAC, EZH2, BET, LSD1, CBP/p300	Restore antigen presentation, enhance IFN response, induce viral mimicry, and suppress EMT/stemness programs	Antigen-presentation-low, IFN-low
**Niche targeting**	TGF-β, VEGF, CSF1R, CCR2/CXCR2, adenosine, CAF/TAM	Disrupt the protective niche that sustains residual tumor cells	CAF/TGFβ-high, TAM/MDSC-rich
**Adaptive adjuvant therapy**	ctDNA/MRD + residual-state profiling	Escalate postoperative therapy based on biological residual states rather than ypTNM/TRG alone	All high-risk residual states
**Ferroptosis induction**	GPX4, System Xc^-^, lysosomal iron (Fento-1), ACSL4	Exploit DTP-acquired antioxidant dependency; selectively kill persister cells via lipid peroxidation	Persister-like/dormant state; stem-like state
**Metabolic synthetic lethality**	FAO (ACOX1, CPT1A/etomoxir), OXPHOS, PERK/ISR, ATR/CHK1, BH3 mimetics (navitoclax)	Exploit metabolic reprogramming, replication stress, and anti-apoptotic dependency unique to DTP cells	Persister-like/dormant state; immune-evasive state (BH3 mimetics for cross-resistance)

## Data Availability

No new data were created or analyzed in this study. Data sharing is not applicable to this article.

## Abbreviations

The following abbreviations are used in this manuscript:

CAFs	Cancer-associated fibroblasts
COX-2	Cyclooxygenase-2
CTC	Circulating tumor cell
ctDNA	Circulating tumor DNA
DAMPs	Damage-associated molecular patterns
DTP	Drug-tolerant persister
EC	Esophageal cancer
ECM	Extracellular matrix
EFS	Event-free survival
EMP	Epithelial–mesenchymal plasticity
EMT	Epithelial–mesenchymal transition
FAO	Fatty acid oxidation
GC	Gastric cancer
GPX4	Glutathione peroxidase 4
HCC	Hepatocellular carcinoma
HDAC	Histone deacetylase
HIF	Hypoxia-inducible factor
HNC	Head and neck cancer
HR	Hazard ratio
HSP	Heat-shock protein
ICI	Immune checkpoint inhibitor
IF	Immunofluorescence
IFN	Interferon
IHC	Immunohistochemistry
IPCs	Immunotherapy persister cells
ISR	Integrated stress response
MDSCs	Myeloid-derived suppressor cells
MRD	Minimal residual disease
NICT	Neoadjuvant immunochemotherapy
NSCLC	Non-small cell lung cancer
OXPHOS	Oxidative phosphorylation
pCR	Pathological complete response
PG	Prostaglandin
OS	Overall survival
RAGE	Receptor for advanced glycation end products
scRNA-seq	Single-cell RNA sequencing
TAMs	Tumor-associated macrophages
TLR	Toll-like receptor
TLS	Tertiary lymphoid structure
TME	Tumor microenvironment
TNBC	Triple-negative breast cancer
Tregs	Regulatory T cells
